# Supplementary material for: Transcriptomic Insights into Mechanisms of Early Seed Maturation in the Garden Pea (Pisum sativum L.)
Source: Cells. 2020 Mar 23;9(3):779. doi: 10.3390/cells9030779 (PMC7140803; doi:10.3390/cells9030779)
Supplement: Supplementary file 1 [file cells-09-00779-s001.zip › Supplementary materials/FileS2.zip!/FileS1/Supporting Maps&Profiles/Supporting Maps.pdf]

Population Map

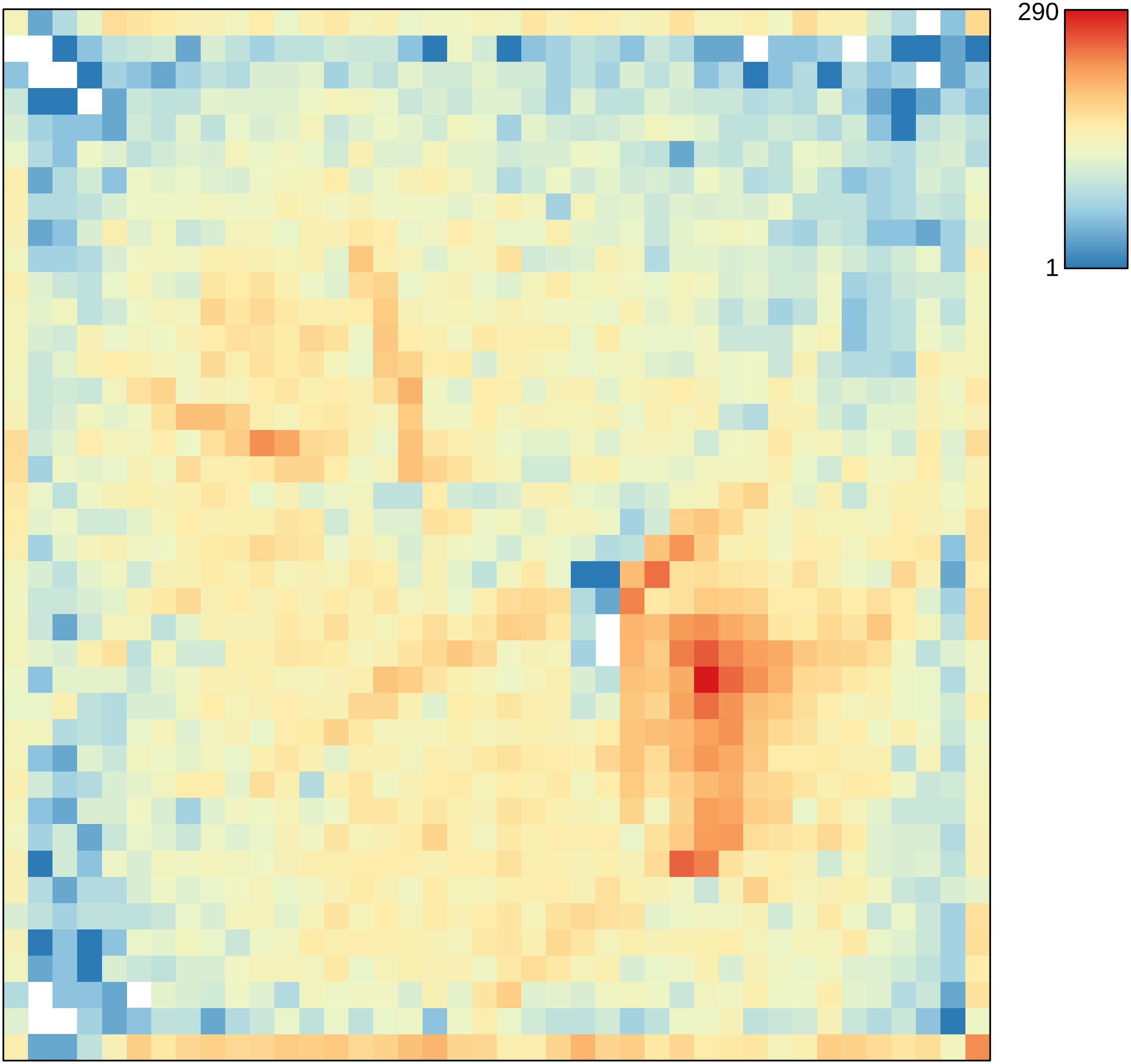

log (# genes in metagene)

Metagene Variance Map

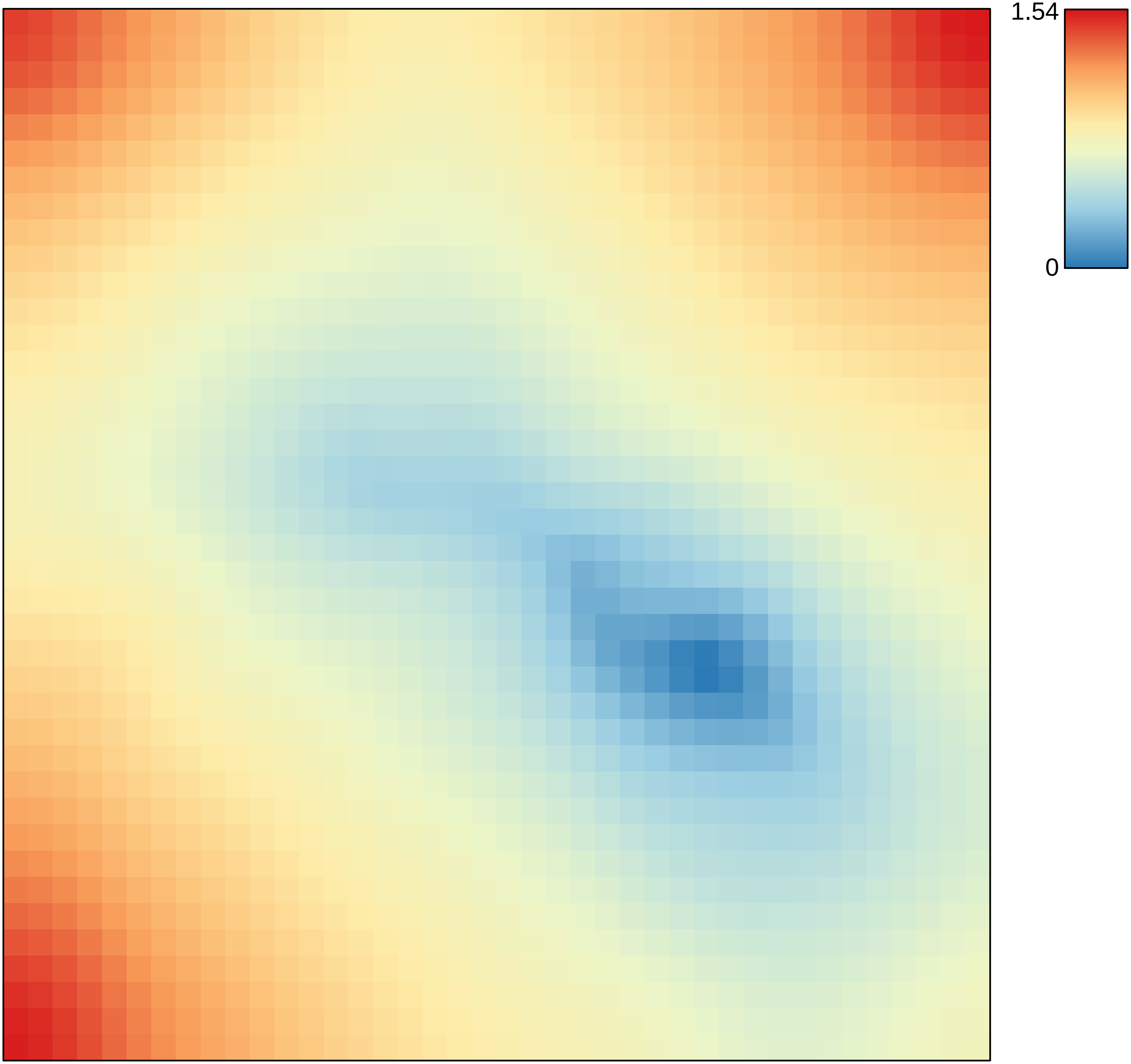

log (metagene variance)

Metagene Significance Map

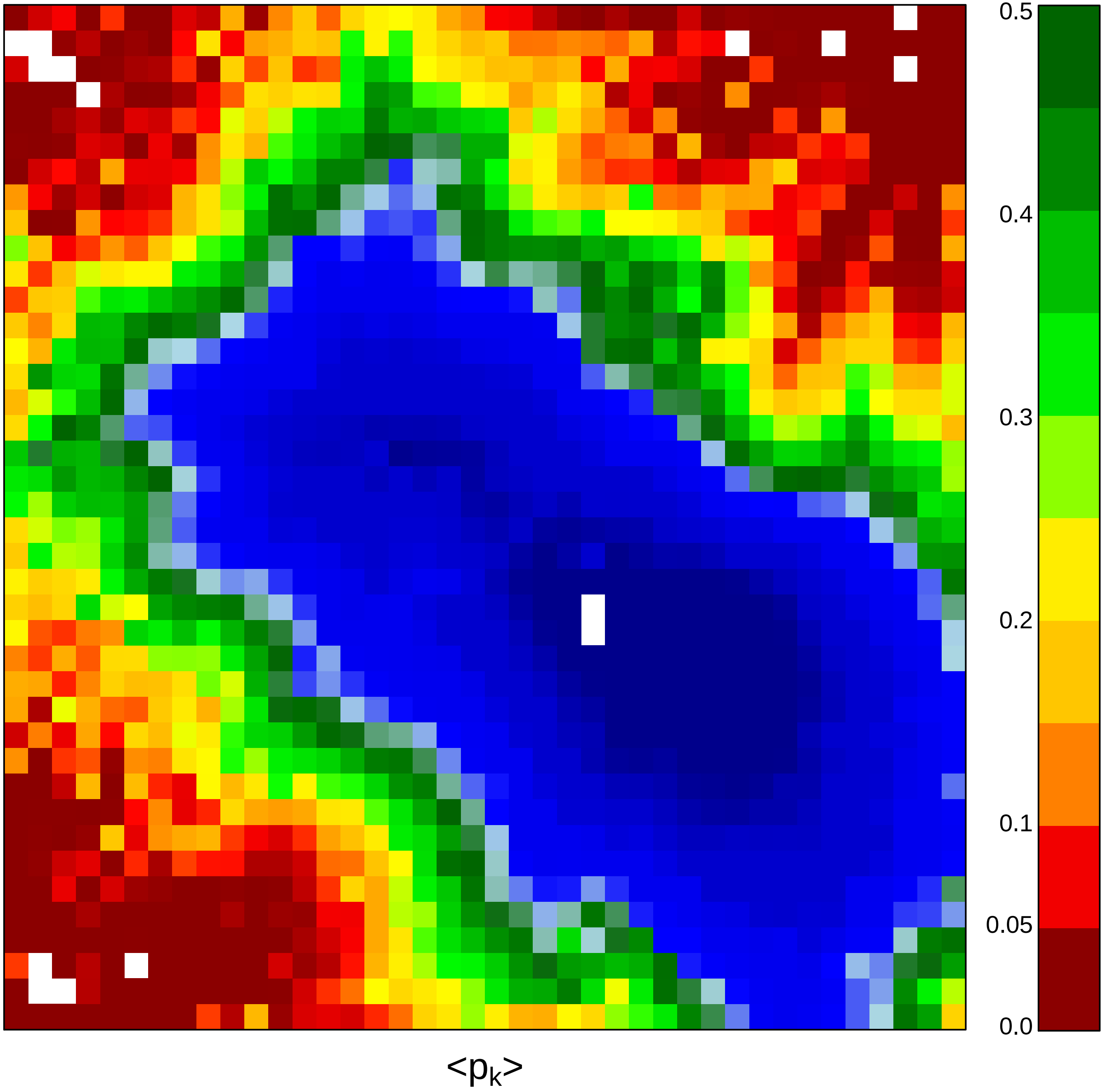

Metagene Significance Map

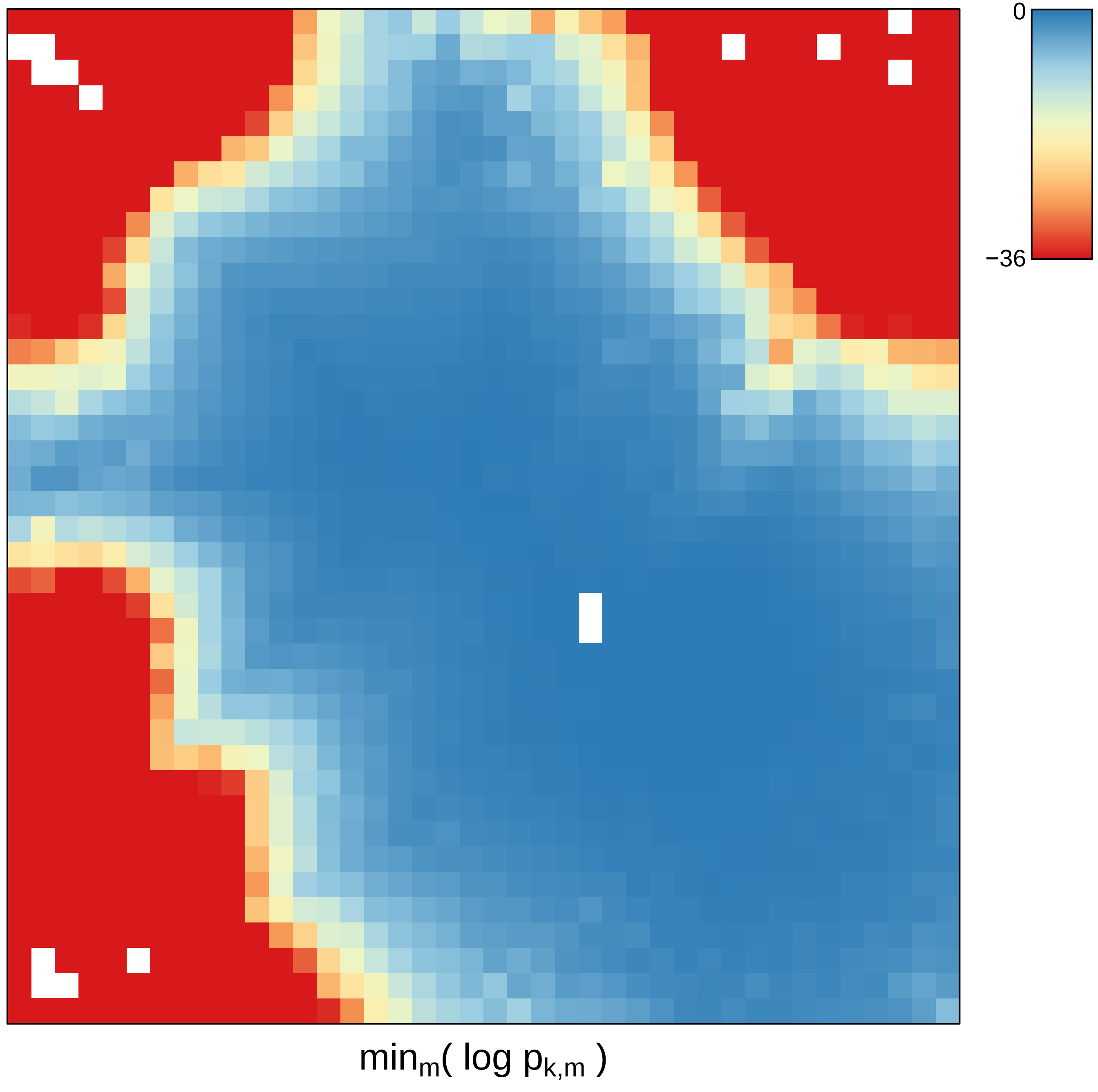

Standard Metagene Entropy Map

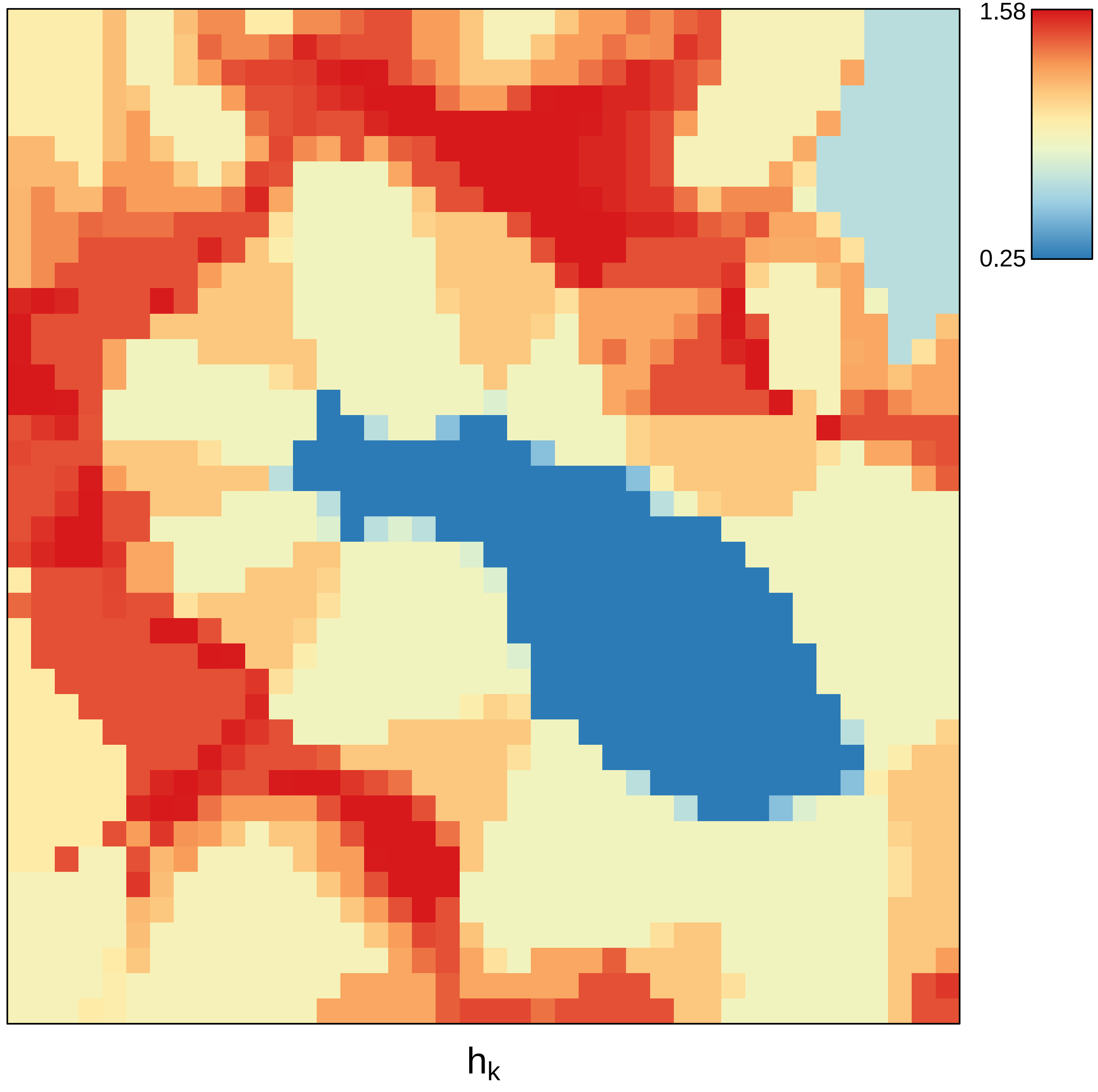

Gene−Metagene Covariance Map

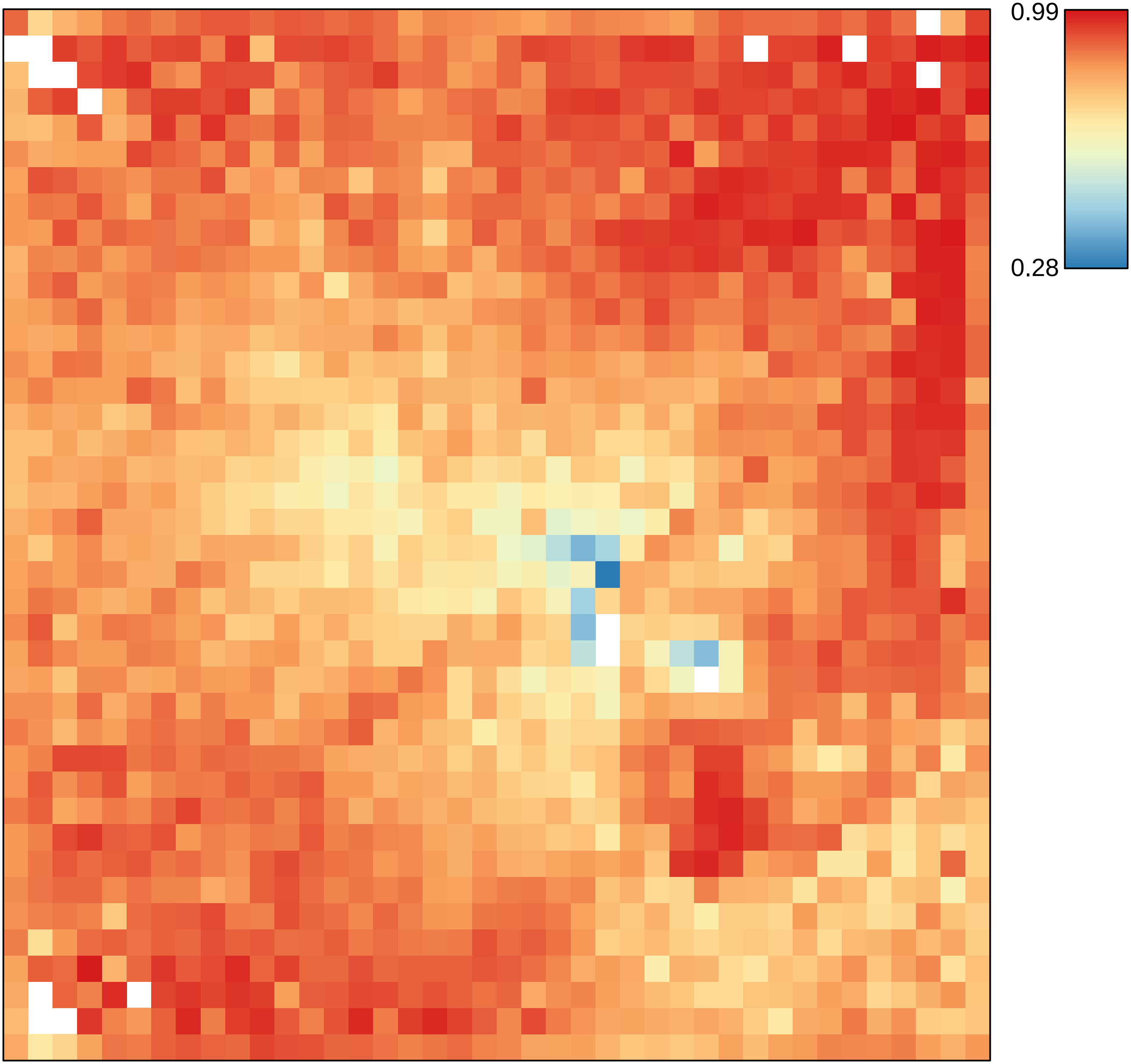

correlation genes − metagene

Deviation Map

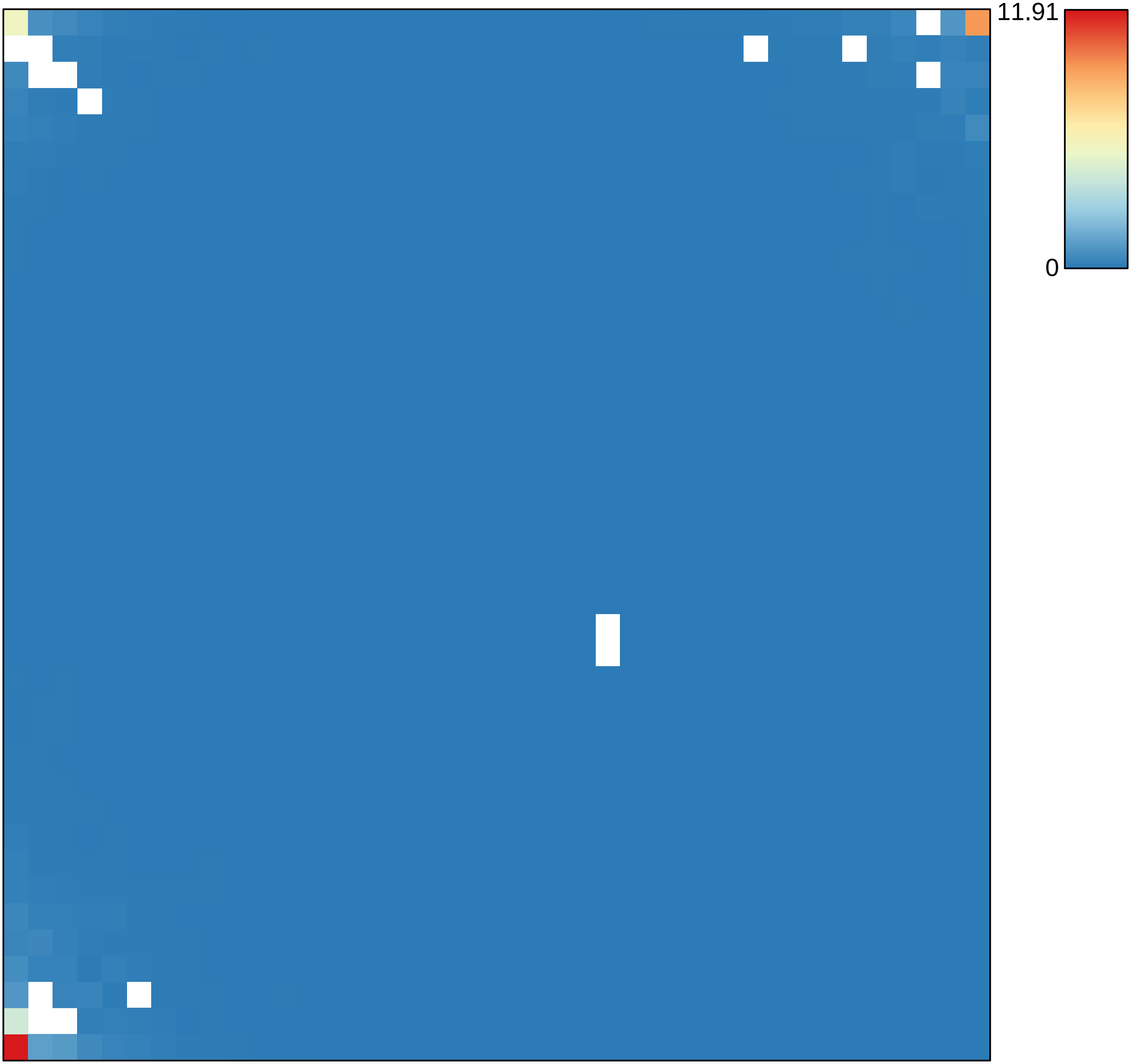

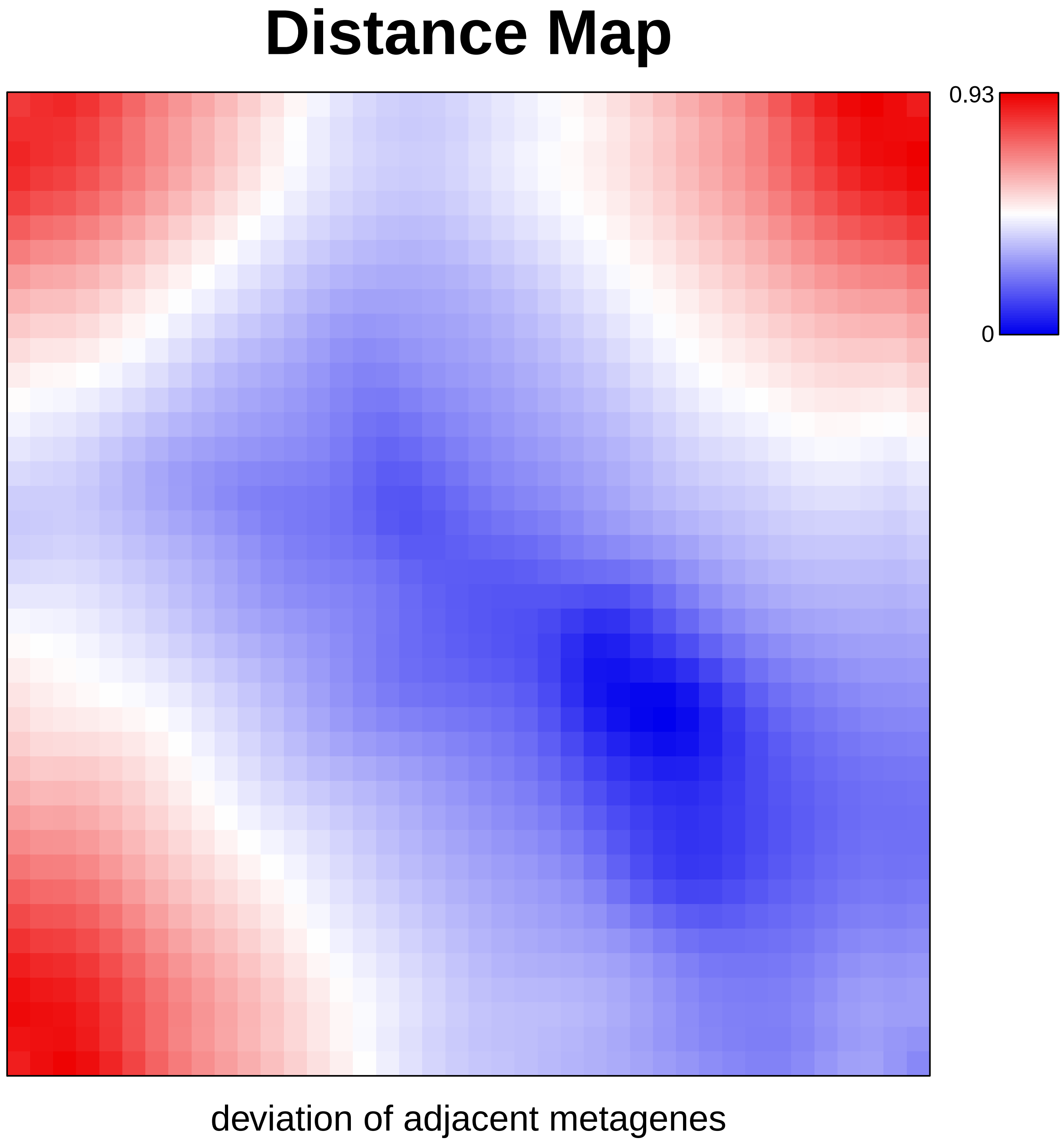
Distance Map
0.93
0
deviation of adjacent metagenes
